# Retrospective investigation of the prognostic value of the β1 integrin expression in patients with head and neck squamous cell carcinoma receiving primary radio(chemo)therapy

**DOI:** 10.1371/journal.pone.0209479

**Published:** 2018-12-20

**Authors:** Nils Cordes, Michael Ney, Thomas Beleites, Daniela Aust, Gustavo Baretton, Howard Thames, Michael Baumann, Mechthild Krause, Steffen Löck, Steffen Appold

**Affiliations:** 1 OncoRay – National Center for Radiation Research in Oncology, Faculty of Medicine and University Hospital Carl Gustav Carus, Technische Universität Dresden, Dresden, Germany; 2 Helmholtz-Zentrum Dresden - Rossendorf, Dresden, Germany; 3 Department of Radiation Oncology, University Hospital Carl Gustav Carus, Technische Universität Dresden, Germany; 4 German Cancer Consortium (DKTK), Dresden, Germany; 5 German Cancer Research Center (DKFZ), Heidelberg, Germany; 6 Helmholtz-Zentrum Dresden - Rossendorf, Institute of Radiation Oncology - OncoRay, Dresden, Germany; 7 Department of Otorhinolaryngology, Head and Neck Surgery, Charité-Universitätsmedizin, Berlin, Germany; 8 Department of Otorhinolaryngology, University Hospital Carl Gustav Carus, Technische Universität Dresden, Germany; 9 Department of Pathology, University Hospital Carl Gustav Carus, TU Dresden, German; Lab of Radiobiology and Experimental Radiooncology, GERMANY

## Abstract

This retrospective study evaluated the expression of β1 integrins and associated proteins as prognostic markers for primary radio(chemo)therapy outcome of patients with locally advanced head and neck squamous cell carcinomas (HNSCC). Tissue microarrays were prepared from 224 HNSCC patients undergoing curative primary radio(chemo)therapy from 1996 to 2005. Staining intensities of β1 integrin and its downstream-proteins FAK, phosphorylated FAK as well as the β1 integrin ECM ligands fibronectin and collagen type-I were determined. Their association to the primary endpoint loco-regional control and the secondary endpoints overall survival and freedom from distant metastasis was analyzed by Cox regression. None of the considered molecular parameters showed a significant association with loco-regional control and freedom from distant metastasis. Patients with p16 positive tumors or tumors with a low intensity of fibronectin showed significantly higher overall survival in univariable regression. In multivariable regression including additional clinical parameters, however, these parameters were not significantly associated with overall survival. Our study in a HNSCC patient cohort treated with primary radio(chemo)therapy does not reveal a prognostic value of β1 integrin expression.

## Introduction

Head and neck squamous cell carcinomas (HNSCC) are among the top 20 cancers worldwide with high risk of loco-regional recurrence and cervical lymph node metastases [1–3]. At time of diagnosis, 50 to 70% of patients present with advanced tumor stage including lymph node metastases (~10% of cases) and distant metastases (~10% of cases) resulting in a 5-year overall survival rate ranging from 10 to 50% [4–6]. Dependent on tumor localization, stage, histology and co-morbidities, different therapeutic approaches are used. Surgery is the treatment of choice at early stages. In case of risk factors/co-morbidities, HNSCC patients receive surgery plus radiochemotherapy, while patients presenting with a more advanced stage but still localized disease receive radiochemotherapy or radiotherapy plus modern targeted drugs such as Cetuximab, an inhibitory antibody for the epidermal growth factor receptor (EGFR), as curative approach [7–12]. Thus, the modern treatment concepts resulted in significant improvement of loco-regional control over the last decades. Hypoxia, human papilloma virus (HPV), p53 and γH2AX have been identified as valuable biomarkers that correlate with outcome of radiotherapy and radiochemotherapy [11,13–19].

However, particularly in HPV-negative patients, intense investigations have been directed to identify targetable pathways associated with radioresistance of tumors [20]. Among them are phosphatidylinositol-3 kinase (PI3K)/mammalian target of rapamycin (mTOR) inhibitors [21,22], PARP-1 inhibitors [23], Src inhibitors [24], STAT inhibitors [25] and anti-programmed death receptor 1 (PD-1) agents [26] currently under investigation in clinical trials (www.clinicaltrials.org). Another potential group of targets for anticancer treatment are integrins, which are overexpressed on HNSCC and are key for HNSCC development, progression and therapy resistance as evidently demonstrated by preclinical, histological and genetic studies [16,27–36].

Integrins are heterodimeric transmembrane receptors for cell adhesion [37]. With their dual functionality for structure and signaling, integrins play a critical role in tissue integrity and cell function control as they channel promitotic and resistance-mediating biochemical cues from the extracellular space [37,38]. Preclinical work exhibited integrin targeting as a promising strategy for radiochemosensitization in various cancer types like HNSCC, breast carcinoma and glioblastoma [39–42]. Among all 24 known integrin receptors composed of an α and a β subunit [37], β1 integrin seems to play the most prominent role through its presence in 12 out of the 24 possible combinations. Extracellular ligands of β1 integrins are extracellular matrix (ECM) proteins like collagens, laminins and fibronectin [43]. Signaling by β1 integrin, similar to other integrin receptors, is facilitated by recruitment of cytoplasmic protein kinases such as focal adhesion kinase (FAK), Src, and Akt [44,45]. In contrast to breast and prostate cancer, FAK has been shown to present the most important determinant downstream of β1 integrin for radiochemoresistance in HNSCC and its phosphorylation status is directly linked to the activation of β1 integrin [39,42,46]. Hence, β1 integrin seems to play a fundamental role in many cancers and its biological function and contribution to therapy resistance are well characterized in HNSCC and other tumor types. Regarding the prognostic value of β1 integrin expression, studies have been performed in breast cancer [47,48], non-small cell lung cancer (NSCLC) [49], in colorectal cancer [50], in early stage glottic laryngeal carcinoma [51], in locally advanced HNSCC [52], and in metastatic versus nonmetastatic primary HNSCC [53] with controversial results for disease-free and overall survival. Concerning the prognostic value of β1 integrin extracellular ligands such as fibronectin, collagens and laminins as well as intracellular signaling mediators such as FAK, a small set of studies revealed a correlation between an upregulation of these proteins or increased copy numbers with HNSCC patients survival [54–56].

Based on these controversial data and the promising observations made for β1 integrin targeting in preclinical HNSCC models [30,42,57,58], we evaluated, for the first time as to our knowledge, the prognostic value of β1 integrin in HNSCC patients receiving primary radiotherapy or radiochemotherapy. In addition, we investigated a selected number of associated adhesion and signaling proteins for which a role for β1 integrin has been demonstrated.

## Materials and methods

### Patient population

The study was approved on 01/22/2008 by the ethics committee of the Medical Faculty, University of Dresden (EK249102007). All data were fully anonymized before usage and informed consent was obtained from each subject, and all procedures were performed in accordance with the Helsinki Declaration. All patients with tumors of the oral cavity, oropharynx, hypopharynx, supraglottic, and larynx who were treated at the Department of Radiation Oncology, University Hospital Carl Gustav Carus, between 01/01/1996 and 12/31/2005 were reviewed. Of these 1137 patients only those were included in the study who fulfilled the following criteria: good general condition at date of diagnosis (WHO 0–2), primary radiotherapy with curative intent to a minimum dose of 68 Gy with or without concurrent chemotherapy, absence of distant metastasis, no second malignancy during the prior five years, complete follow up and histologic specimen available. Retrospective clinical data such as tumor stage according to the 7^th^ edition of the TNM classification [59], primary tumor site, dose and duration of radiochemotherapy, age, gender, smoking as well as histopathological data such as histological grading were recorded. Finally, the statistical analysis was based on 224 patients. Of these, 123 (54.9%) were treated with chemotherapy using 5-fluoruracil (5-FU; 600 mg/m^2^; day 1–5) and cisplatin (30 mg/m^2^ weekly). Patients were followed retrospectively for up to 10 years.

### Tissue microarrays and tissue specimens

Tissue microarrays (TMA) have been produced using a manual arrayer (MTA-1, Alpha Metrix, Germany). With a hollow needle, tumor tissue cores with a diameter of 0.6 mm obtained from paraffin embedded primary tumor were inserted in the recipient paraffin block. From each patient, three tumor tissue cores were included in the TMA.

### Immunohistochemistry

Histological sections (4 μm) were obtained from each TMA using a microtome (Leica, Germany). Each slide was heated up to 60°C for 15 minutes, deparaffinated in Xylene and rehydrated through graded alcohols. The slides were placed in methanol/30% hydrogen peroxide for 20 minutes, microwaved for 3.5 minutes in 10 mmol/l sodium citrate three times. Interim cool down was accomplished at room temperature for 20 minutes (heat induced epitope retrieval, HIER). Sections were blocked using an avidine/biotin blocking kit (Vectastain ABC Biologo, Kronhagen, Germany) followed by antibody incubation at room temperature overnight. The following primary antibodies were used: anti-β1 integrin (Calbiochem, CP26-100UG, 1:10), anti-collagen I (Rockland/Biomol, 600-401-103-0, 1:50), anti-fibronectin (FN, BD Bioscience, 61077, 1:100), anti-FAK (Millipore, 06–543, 1:50), anti-p-FAK (Invitrogen, 44624G, 1:200), anti-Ki-67/MIB1 (Dako, OP43, 1:500), anti-p53 (Calbiochem, M7240, 1:2000) and anti-p16^INK4^ (E6H4) (CINtec/Roche, 9517, ready to use). Antibody detection was done using the avidine-biotinylated enzyme complex (Vectastain ABC Biologo, Kronhagen, Germany) followed by antibody visualization by incubation with the chromogene diaminobenzidine (DAB) in absolute darkness for ten minutes and counterstaining with hematoxylin. After dehydration through graded alcohols and incubation in xylene for five minutes, slides were covered air tight.

### Scoring

The expression levels of stained proteins were evaluated based on their staining intensity for each of the tumor tissue cores. Staining intensities were defined as: negative = 0, weak = 1, moderate = 2, strong = 3 as previously published [60]. All sections were scored by two experienced pathologists in a blinded fashion using a transmitted-light binocular microscope (Olympus BH-2, Olympus, Hamburg, Germany). Specimens with insufficient amount of core tumor tissue, disruption or loss of the tissue were excluded. At least two of the three cores per patient had to show tumor tissue. The intensities of the different cores were combined to a final intensity score using their median value. The following classification scheme was applied: median 0 = intensity 0, median 0.5–1.5 = intensity 1, median 2–2.5 = intensity 2 and median 3 = intensity 3.

The evaluation of staining patterns of Ki-67/MIB1, p53 and p16 was done according to the percentage of tumor cells expressing the marker. Expression of Ki-67/MIB1 and p53 in more than 10% of tumor cells was assessed as positive. Expression of p16 in 70% and more tumor cells per core was considered positive. Blinded samples were scored by two independent observers (MN and NC) with an inter-observer variability of <5%.

### Statistical analysis

Primary endpoint of the study was loco-regional control (LRC) and secondary endpoints were overall survival (OS) and freedom from distant metastasis (FDM). Survival times were calculated from the start of radiation therapy to the respective event (local or regional recurrence for LRC, death for OS and distant metastasis for FDM) or censoring. Survival curves were estimated by the Kaplan-Meier method and compared between subgroups by the log-rank test. To investigate the association of clinical and molecular parameters with the defined endpoints, univariable Cox proportional hazards regressions were performed. Significant clinical parameters were combined with one molecular parameter in multivariable regression models. For the molecular parameters given by intensity values between 0 and 3, an overall chi-squared test was performed within Cox regression to identify potential differences in survival between any of the four groups. If at least a statistical trend was observed, patients were stratified in two groups based on an intensity cutoff. Correlations between categorical variables were evaluated by the Spearman correlation coefficient (ρ). Statistical analyses were performed using IBM SPSS Statistics 25 (IBM Corporation, Armonk, NY). For all analyses, two-sided tests were performed and p-values < 0.05 were considered statistically significant.

## Results

**Fig 1:**
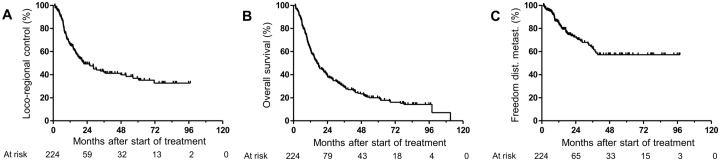
Impact of β1 integrin expression on different endpoints. Kaplan-Meier curves of (A) loco-regional control, (B) overall survival and (C) freedom from distant metastases for all patients.

Characteristics of the 224 included patients are presented in Table 1. The oropharynx was the most common primary tumor site in the evaluated patient population. Eighty-eight (39.3%) of the HNSCC were localized at the oropharynx, 70 (31.2%) at the hypopharynx, 51 (22.8%) at the oral cavity and 15 (6.7%) at the supraglottic larynx. According to the 7^th^ edition of the TNM classification [59], 154 patients (68.8%) had a T4 tumor stage whereas 32 (14.3%) featured cT3, 29 (12.9%) cT2 and only 8 (3.6%) cT1 tumor stage at time of tumor diagnosis. Moreover, 205 patients (91.5%) presented with cervical lymph node metastases in which most of them (164, 73.2%) had a cN2 stage. LRC after two and five years was 49.3% and 36.8%, respectively (Fig 1A), while OS after two and five years was 39.2% and 19.9%, respectively (Fig 1B). Median follow-up was 55.1 months (range: 1.4–97.1 months) for patients alive at the time of analysis (47/224 patients).

**Table 1:** Patient characteristics.

**Variable**		**of 224**	**Fraction (%)**
Gender	Male / Female	196 / 28	87.5 / 12.5
Tumor localization	Oral cavity / Oropharynx	51 / 88	22.8 / 39.3
	Hypopharynx, Supraglottic larynx	70 / 15	31.2 / 6.7
cT stage	1 / 2	8 / 29	3.6 / 12.9
	3 / 4 / Missing	32 / 154 / 1	14.3 / 68.8 / 0.4
cN stage	0 / 1	18 / 11	8.0 / 4.9
	2 / 3	164 / 30	73.2 / 13.4
	Missing	1	0.5
Grade	1 / 2	6 / 145	2.7 / 64.7
	3 / 4	64 / 2	28.6 / 0.9
	Missing	7	3.1
WHO	0 / 1	17 / 181	7.6 / 80.8
	2 / 3	21 / 5	9.4 / 2.2
Smoking	Never / Yes	17 / 170	7.6 / 75.9
	Stopped / Missing	29 / 8	12.9 / 3.6
Chemotherapy	Yes	123	54.9
	No	101	45.1
p53	Negative	108	48.2
	Positive	113	50.5
	Missing	3	1.3
p16	Negative	180	80.3
	Positive	38	17.0
	Missing	6	2.7
**Variable**	**Median (Range)**		
Age	58.5 (35.3–91.8)		
Delivered dose (Gy)	70.6 (68.0–77.6)		
Treatment time (days)	40.5 (36.0–58.0)		

Abbreviations: WHO, World Health organization

Patients with cN3 showed significantly lower LRC (p = 0.002) and patients with p16 positive tumors a statistical trend (p = 0.072) towards higher LRC in univariable regression. Significantly higher OS was obtained for patients with low cN stage (p = 0.002), supraglottic tumors (p = 0.020), low WHO stage (p = 0.001), non-smokers (p = 0.005), p16 positive tumors (p = 0.005) and short treatment time (p = 0.048). Only cN stage was significantly related to FDM (p<0.001). The use of chemotherapy had no significant impact on the considered endpoints and was not significantly correlated with the protein expression intensities. Results of univariable analysis are presented in Table 2. In addition, we performed Cox regressions separately for patients with and without chemotherapy (data not shown). Similar results were obtained for both groups, except for Ki-67/MIB1, which was significantly associated with reduced DM in the group treated by radiochemotherapy (p = 0.033) but not in the group treated by radiotherapy only.

**Table 2:** Univariable Cox regression of loco-regional control, overall survival and freedom from distant metastases. For the β1 integrin related parameters only results with at least a statistical trend (p<0.1) for one endpoint are reported (Fibronectin and Collagen type-I).

	Loco-regional control		Overall survival		Freedom from distant metastases	
Variable	HR (95% CI)	p-value	HR (95% CI)	p-value	HR (95% CI)	p-value
Gender (male vs female)	0.76 (0.41–1.39)	0.38	0.78 (0.49–1.24)	0.29	0.67 (0.29–1.58)	0.36
Age (years)	0.99 (0.97–1.01)	0.47	0.99 (0.98–1.01)	0.33	0.98 (0.95–1.00)	0.090
Location (overall)	*	0.22	*	0.058	*	0.89
Location (others vs supraglottic)	0.47 (0.19–1.15)	0.098	0.43 (0.21–0.88)	**0.020**	0.78 (0.28–2.15)	0.63
cT (0–2 vs 3–4)	1.40 (0.81–2.44)	0.23	1.17 (0.79–1.73)	0.45	0.90 (0.46–1.75)	0.75
cN (0–2 vs 3)	2.46 (1.39–4.38)	**0.002**	2.02 (1.31–3.14)	**0.002**	3.75 (1.86–7.55)	**<0.001**
Grade (1,2 vs 3,4)	0.80 (0.51–1.27)	0.35	0.82 (0.58–1.14)	0.24	0.69 (0.36–1.32)	0.26
WHO status (0,1 vs 2,3)	1.52 (0.82–2.79)	0.18	2.03 (1.32–3.12)	**0.001**	1.38 (0.55–3.50)	0.50
Smoking (never vs others)	1.98 (0.87–4.55)	0.11	2.63 (1.39–5.17)	**0.005**	1.98 (0.61–6.37)	0.25
Chemotherapy	0.96 (0.64–1.43)	0.96	0.82 (0.61–1.10)	0.18	1.30 (0.74–2.29)	0.37
Dose (Gy)	0.98 (0.92–1.06)	0.64	1.03 (0.97–1.08)	0.34	0.99 (0.90–1.09)	0.85
Treatment time (days)	1.03 (0.97–1.09)	0.33	1.04 (1.00–1.09)	**0.048**	1.01 (0.93–1.10)	0.75
p53 (negative vs positive)	1.22 (0.82–1.83)	0.33	1.20 (0.89–1.64)	0.24	0.72 (0.41–1.25)	0.24
p16 (negative vs positive)	0.58 (0.33–1.05)	0.072	0.52 (0.33–0.83)	**0.005**	0.75 (0.35–1.59)	0.45
Fibronectin (0 vs 1–3)	5.57 (0.76–39.3)	0.092	3.14 (1.00–9.85)	**0.050**	1.02 (0.31–3.29)	0.98
Collagen type-I (0 vs 1–3)	1.36 (0.81–2.30)	0.25	1.41 (0.97–2.06)	0.072	0.50 (0.18–1.38)	0.18

Abbreviations: WHO, World Health Organization; HR = hazard ratio; 95% CI = 95 percent confidence interval

*overall test

**Fig 2:**
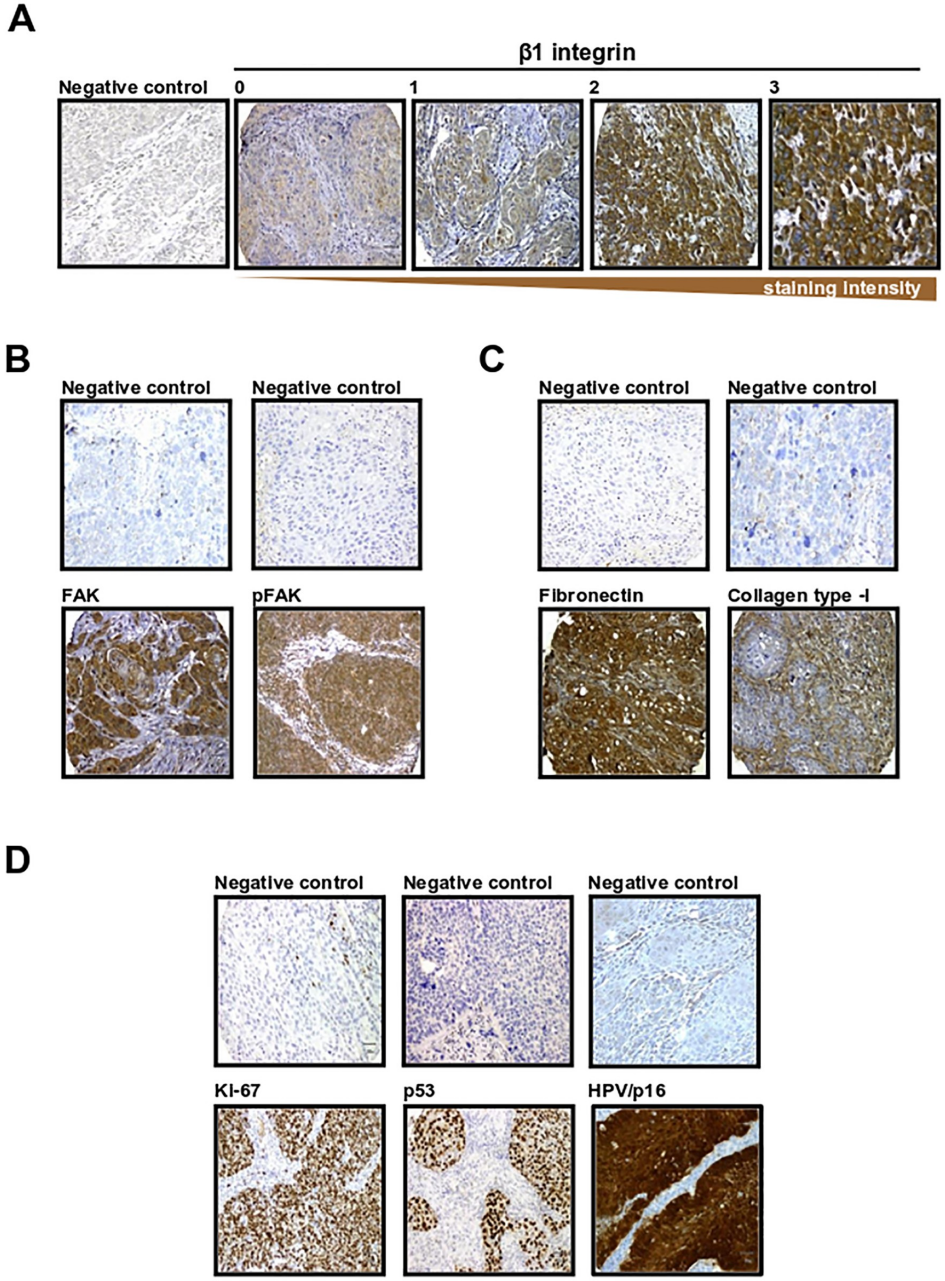
Immunohistochemical staining of β1 integrin and associated proteins. Representative immunohistochemical staining of β1 integrin (A), FAK and phosphorylated FAK (pFAK) (B), associated ECM proteins (C), proliferation markers and HPV/p16 (D) in HNSCC specimens, including negative controls. Immunochemistry was performed on paraffin embedded tissue microarrays (TMA). A shows the four different β1 integrin expression scores (0 = absent; 1–3 increasing. B shows only score 3 stainings relative to the negative control. Magnification is x20 for all photographs.

The expression intensities of β1 integrin and associated proteins as well as p53, Ki-67/MIB1 and p16 are shown in Table 3. In general, we found in our immunohistochemical staining for β1 integrin (Fig 2A), FAK and pFAK a membranous/cytoplasmic localized pattern (Fig 2B), for the ECM proteins FN and Col-I an extra-/intracellular pattern (Fig 2C), and for p53 and Ki-67/MIB1 a strong nuclear pattern (Fig 2D). FN and Col-I were expressed extra- and intracellularly. While FN showed localization in all tumor areas, Col-I expression was only found in some tumor islands, which requires further investigation. Correlations of these expression intensities with clinical parameters were generally low. The highest correlations were observed between Ki-67/MIB1 intensity and N stage (ρ = 0.21, p = 0.002) as well as smoking status (ρ = 0.21, p = 0.003). Furthermore, the intensities of β1 integrin, FAK and pFAK were significantly but weakly correlated with each other (ρ<0.3, p≤0.001; Table 4).

**Table 3:** Expression of proteins of interest in HNSCC patients.

Intensity		0		1		2		3	
**Variable**	**n**	**n**	**%**	**n**	**%**	**n**	**%**	**n**	**%**
β1 integrin	220	5	2.3	86	39.1	117	53.2	12	5.5
FAK	221	1	0.5	72	32.6	135	61.1	13	5.9
pFAK	223	35	15.7	140	62.8	44	19.7	4	1.8
Fibronectin	221	7	3.2	86	38.9	102	46.2	26	11.8
Collagen-I	221	179	81.0	30	13.6	12	5.4	0	0
		**Negative**						**Positive**	
				**n**	**%**			**n**	**%**
Ki67/MIB	219			72	32.9			147	67.1
P53	221			108	48.9			113	51.1
p16	218			181	83.0			37	17.0

Abbreviations: FAK, focal adhesion kinase; pFAK, phosphorylated FAK; MIB, Molecular Immunology Borstel.

**Table 4:** Cross tables for the intensities of β1 integrin, FAK and pFAK and results of Spearman correlation analyses.

		**β1 integrin intensity**				**FAK intensity**			
		**0**	**1**	**2**	**3**	**0**	**1**	**2**	**3**
**pFAK intensity**	**0**	4	13	16	1	1	17	15	1
	**1**	1	60	76	1	0	48	86	6
	**2**	0	11	23	9	0	6	31	5
	**3**	0	2	2	0	0	1	2	1
				ρ = 0.22, p = 0.001				ρ = 0.26, p<0.001	
**FAK intensity**	**0**	1	0	0	0				
	**1**	4	35	31	1				
	**2**	0	49	75	10				
	**3**	0	2	10	1				
				ρ = 0.26, p<0.001					

Abbreviations: FAK, focal adhesion kinase; pFAK, phosphorylated FAK; ρ, Spearman correlation coefficient.

**Fig 3:**
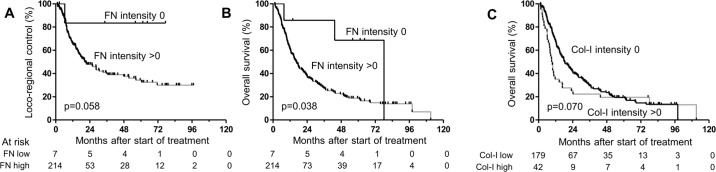
Patient survival and marker expression. Kaplan-Meier curves of overall survival for patients stratified by (A) FN intensity (0 vs > 0) and (B) Col-I intensity (0 vs > 0). p-values originate from log-rank tests.

We analyzed staining patterns of β1 integrin, FAK, pFAK, FN and Col-I for associations with the considered endpoints. Only FN (intensity 0 vs others) showed a statistical trend for LRC in univariable analysis, where intensity 0 was related to higher LRC (p = 0.092, Fig 3A). For OS, FN intensity 0 led to significantly higher OS compared to higher intensities in univariable regression (p = 0.050, Fig 3B), while Col-I intensity 0 showed a statistical trend (p = 0.072, Fig 3C). Only 7 patients were in the FN-negative group. Multivariable regression for LRC including cN stage and FN intensity (0 vs others) confirmed the statistical trend for FN (p = 0.067). Multivariable regression for OS, including cN stage, tumor location, WHO stage, smoking status, p16 status, treatment time and FN intensity (0 vs others) or Col-I intensity (0 vs others) was unable to confirm the univariable results (p = 0.13 and p = 0.15, respectively; Table 5).

**Table 5:** Multivariable Cox regression of loco-regional control (LRC) and overall survival (OS). One β1 integrin-related parameter, which showed a statistical trend with LRC or OS in univariable analyses (Fibronectin or Collagen type-I), was combined with cN stage for LRC and with tumor location, cN stage, WHO status, smoking status, treatment time and p16 status for OS.

Variable	HR (95% CI)	p-value
**Loco-regional control**		
cN (0–2 vs 3)	2.68 (1.50–4.78)	**0.001**
Fibronectin (0 vs 1–3)	6.36 (0.88–45.9)	0.067
**Overall survival**		
Location (others vs supraglottic)	0.39 (0.18–0.84)	**0.016**
cN (0–2 vs 3)	1.90 (1.19–3.04)	**0.008**
WHO status (0,1 vs 2,3)	1.74 (1.11–2.73)	**0.015**
Smoking (never vs others)	2.77 (1.23–6.26)	**0.014**
Treatment time (days)	1.03 (0.99–1.08)	0.18
p16 (negative vs positive)	0.67 (0.41–1.08)	0.11
Fibronectin (0 vs 1–3)	2.97 (0.73–12.1)	0.13
Location (others vs supraglottic)	0.40 (0.19–0.87)	**0.021**
cN (0–2 vs 3)	2.03 (1.27–3.27)	**0.003**
WHO status (0,1 vs 2,3)	1.75 (1.12–2.74)	**0.015**
Smoking (never vs others)	3.09 (1.38–6.91)	**0.006**
Treatment time (days)	1.02 (0.97–1.07)	0.39
p16 (negative vs positive)	0.65 (0.40–1.05)	0.080
Collagen type-I (0 vs 1–3)	1.34 (0.90–2.01)	0.15

Abbreviations: WHO, World health Organization; HR = hazard ratio; 95% CI = 95 percent confidence interval

## Discussion

Tumor cell-ECM interactions confer a survival advantage through enhanced prosurvival signaling. Consequently, tumor cells show higher intrinsic resistance to standard therapies such as radiotherapy and chemotherapy as well as increased levels of local infiltration and metastasis. Our increasing knowledge about these factors not only promotes the development of molecular-targeted therapies to disrupt cell-ECM interactions but also offers new possibilities to identify new biomarkers. The present study evaluated the prognostic value of β1 integrins in HNSCC patients receiving primary radio(chemo)therapy. We found that (i) β1 integrin and its downstream proteins FAK, pFAK as well as the β1 integrin ECM ligands FN and Col-I were differentially expressed in HNSCC, (ii) the expression of β1 integrin, FAK, pFAK, Col-I and p53, at least under the statistical limitations of low event numbers in our study, failed to provide a statistically significant connection with loco-regional control, overall survival and freedom from distant metastasis, (iii) patients with p16-positive tumors or tumors with a low intensity of fibronectin showed significantly higher overall survival in univariable regression; in multivariable regression including additional clinical parameters, however, these parameters were not significantly associated with overall survival.

Due to the fact that the preclinical evaluation of β1 integrin targeting yielded a large body of promising observations, studies were conducted to elucidate the prognostic value of the expression of β1 integrins and its associated proteins in retrospective genetic and/or histological studies in breast carcinomas [40,47,48,61], NSCLC [49], small cell lung cancer [62], colorectal liver metastasis [63], the early stage glottic carcinomas [51], and in locally advanced HNSCC [52].

In early T1/T2 stage glottis carcinomas, Choi and colleagues showed a higher risk for local tumor recurrence with a strong and diffuse β1 integrin expression compared with HNSCC with a more localized or missing β1 integrin expression [51]. Similarly, Koukourakis and colleagues found membranous/cytoplasmic localization of β1 integrin in locally advanced HNSCC [52], which is in line with our results. Further, they observed the putative cancer stem cell markers CD44 and Oct4 as well as β1 integrin to be associated with poor prognosis. In their multivariate analyses, Koukourakis and colleagues report β1 integrin to have independent statistical significance with regards to local relapse, distant metastases and overall survival.

In contrast to small cell lung cancer [62], β1 integrin expression was shown to be highly significantly correlated with a poorer overall survival in NSCLC [62]. By means of mRNA expression profiling, Dingemans and colleagues were able to show that the expression levels of α5, β1 and β3 integrins predict overall and disease-free survival of patients with early stage NSCLC [49]. In liver metastasis of colorectal carcinomas, β1 integrin expression trended with disease-free and overall survival [63]. Interestingly, our data are in clear contrast to data presented by Wang and colleagues who demonstrated that β1 integrin has a strong impact on HNSCC prognosis through participation in the metastatic process [64]. Further studies indicating a prognostic value of β1 integrin for overall survival have been conducted in serous adenocarcinomas of the ovary [65], metastatic melanoma [66], adenocarcinoma of Barrett’s esophagus [67], and periampullary carcinoma but not ductal adenocarcinoma of the pancreas [68].

When reviewing these studies, a critical point is the heterogeneity of the investigated patient populations. In the majority of the studies performed to date, this aspect is further complicated by the heterogeneity of the applied therapy. In comparison, our study included a patient collective with locally advanced HNSCC, i.e. a cohort that was negatively selected regarding local tumor stage (cT3, cT4) and occurrence of lymph node metastases (cN2, cN3). Furthermore, we focused on one treatment strategy and included only patients who completed curatively intended radiotherapy or radiochemotherapy to a radiation dose of at least 66 Gy.

Overall, the data presented in this study indicate no potential for β1 integrin as a prognostic marker. However, as 97.3% of locally advanced HNSCC patients in our cohort treated with curatively intended radio(chemo)therapy express low to high levels of β1 integrin, the potential for β1 integrin as therapeutic target remains and warrants further in-depth investigations. Based on the high prevalence of β1 integrin as well as its downstream target FAK (99.5%; including a low to high phosphorylation status ranging from 62.8% to 1.8%), a rationale for clinical testing of drugs directed against β1 integrin is clearly given.

Intriguing to us were also the expression levels of fibronectin (96.7% positive) and collagen type-I (19% positive). Both proteins were expressed extra- and intracellularly. Fibronectin showed localization in all tumor areas, while collagen type-I lacked expression in some tumor islands, which requires further investigation. Despite our low patient numbers for absent fibronectin expression, increasing levels of fibronectin and collagen type-I significantly correlated with lower patient overall survival in univariable analysis. This is in line with a study by Wang and colleagues reporting β1 integrin expression to be significantly higher in metastatic versus nonmetastatic primary HNSCC [53] and by Misawa and colleagues documenting upregulated mRNA levels of the β1 integrin ligands collagen type-22A1 and 24A1 over the course of HNSCC progression to have prognostic value in HNSCC patients [54]. Another study revealed ECM proteins such as collagens, fibronectin and laminins, again, all β1 integrin ligands, significantly overexpressed in HNSCC versus normal tissue [55]. Concerning the β1 integrin signaling mediator FAK, recent investigations demonstrated FAK copy number to be associated with disease recurrence in HPV-negative HNSCC [56] and FAK deactivation to elicit radiochemosensitization on HNSCC cells [46,56].

Nonetheless, we acknowledge the potential impairments of investigating a novel potential biomarker in clinical tissue specimens. Despite the fact that immunoreactivity of a specific receptor or molecule may be associated with outcome, it may not reflect the biological activity of the receptor or molecule. In addition, although the present cohort has significant follow-up time, the relatively modest sample size necessitates validation studies using larger numbers of patients and matched pairs of patients in large-scale multicenter networks. If such studies reveal prognostic relevance of β1 integrins or their downstream proteins, the rationale may be given for testing drugs directed against resistance pathways of β1 integrins combined with radiation in an integrin positive subset of patients with locally advanced HNSCC undergoing radiochemotherapy.

In conclusion, the data presented in this study indicate no potential for β1 integrin as a prognostic marker in HNSCC patients treated with radio(chemo)therapy. Reasons for this result are likely to lie in the selection criteria for the investigated cohorts, in particular tumor stage, tumor site, tumor entity, the applied therapy and putative inhomogeneities in the tumor tissues. Future prospective histopathological examinations with inclusion of additional criteria and specific focus on phosphorylated FAK are warranted to determine a prognostic as well as a predictive biomarker signature that can reliably be used to individually select HNSCC patients for therapy.
